# Total Closed Talar Dislocation without Ankle Fracture: A Case Report

**DOI:** 10.1055/s-0043-1776295

**Published:** 2024-04-19

**Authors:** Rubén Daniel Arellano, Daniel Orlando Arellano, Diego Fernando Arellano, José Ángel Becerra, José Arturo Ramírez

**Affiliations:** 1 Faculdade de Medicina Torreón, Universidad Autónoma de Coahuila, Torreón Coahuila, México

**Keywords:** ankle fractures/diagnosis, joint dislocations, subtalar joint

## Abstract

We report a case of a 61-year-old female who presented to the emergency room after a fall from stairs. A total closed talar dislocation without talus or ankle fracture was diagnosed. The treating surgeon indicated an open reduction after an unsuccessful attempt at closed reduction. After six months of follow-up, the patient reported mild pain and partial weight-bearing with no discomfort; however, signs of talar avascular necrosis were present on magnetic resonance images and CT scans.

## Introduction

Total talar dislocations (TTDs) are rare injuries accounting for only 0.06% of all dislocations and 2% of talar injuries. 1 They are often the result of high-energy trauma, and almost all cases are of open injuries. Weston et al. 2 reported a frequency of open talar dislocations of 85% and of closed dislocations of 15%. Additionally, this injury can be associated with fractures involving the talus, 3 malleolus, 4 among others. However, closed TTDs without concomitant malleolar and talar fracture occur much less frequently. 5

Avascular necrosis (AVN), osteoarthritis, and infection are the most common complications affecting the prognosis of TTD due to the damage to the talar blood supply and surrounding tissues. Infection is more common following open dislocations and open reduction of an associated talar fracture. 6

Different authors 7 8 have recommended open reduction for TTD; however, the closed reduction has been strongly recommended for closed talar dislocations to avoid injury to soft tissue and damage to the blood supply network. 9

We herein present the case of a patient who sustained a closed, rotated TTD without malleolar or talar fracture.

## Case Report

**Fig. 1:**
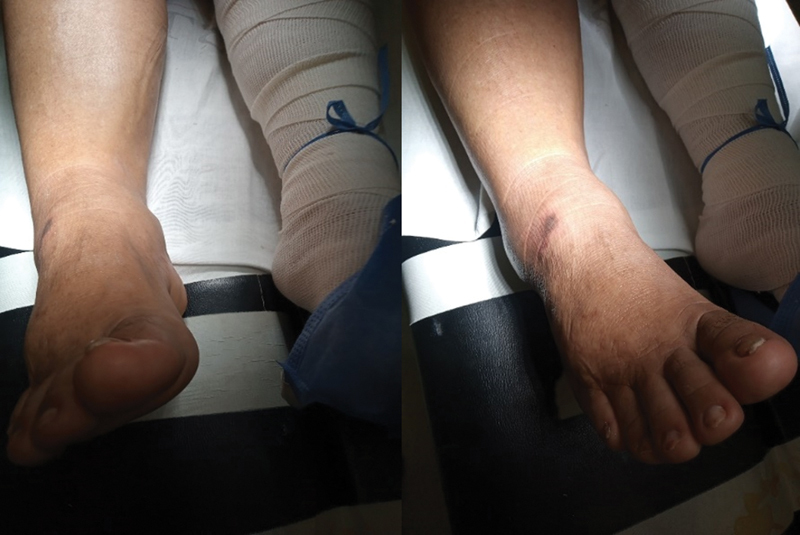
Clinical image showing edema of the ankle and skin lesion.

A 61-year-old female patient presented to the emergency department after slipping while climbing stairs. She complained about pain, swelling, and deformity of her right ankle. Additionally, she had type-II diabetes and liver cirrhosis. The physical examination revealed swelling over the right foot and ankle, varus deformity of the forefoot, and intense pain. The skin had a small scratch without active bleeding; however, no wounds exposing deep tissue were present, nor evidence of current neurological or vascular compromise ( Fig. 1 ).

**Fig. 2:**
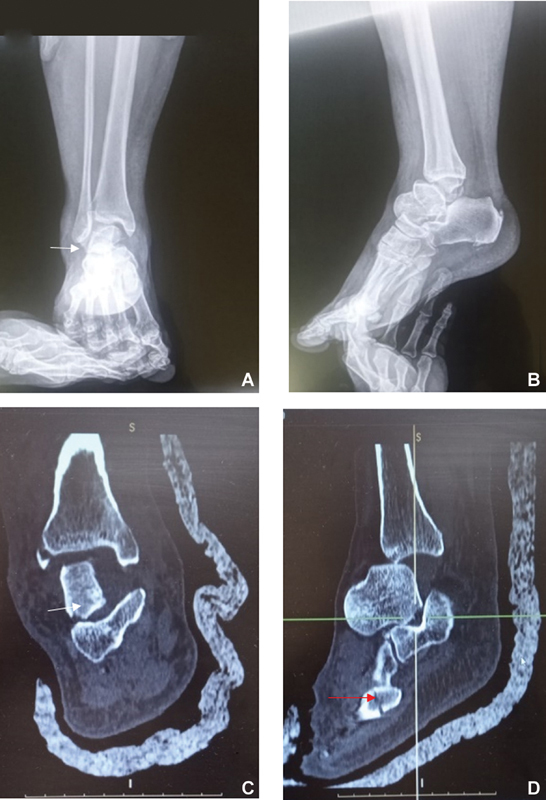
Plain radiographs and computed tomography scans showing total talar dislocation. ( **A,B** ) The white arrows point to the subtalar joint area due to rotation of the talus. The red arrow points to the fracture at the base of the fifth metatarsal bone.

A radiograph and computed tomography (CT) scan of the ankle and foot revealed total anterolateral dislocation of the talus. The talus was rotated medially 90° in the coronal plane, which made the subtalar articular facets point to the lateral malleolus. A fracture without dislocation of the base of the fifth metatarsal was also present. There were no associated fractures of the talus, calcaneus, nor of the medial, lateral, or posterior malleolus ( Fig. 2 ).

**Fig. 3:**
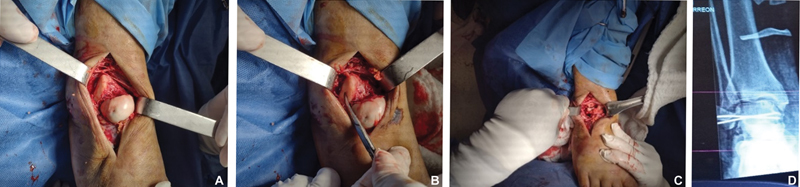
( **A-C** ) Operative images. The surgical forceps points to the subtalar joint area and relocation of the talus. ( **D-F** ) Postoperative radiographs. Notice the osteochondral fracture on the talar dome (red arrow).

Upon admission, an attempt at closed manipulation and reduction was made; however, it was unsuccessful. The patient was then admitted to the hospital and scheduled for an open reduction surgery of the talus. This procedure was successful. Surgery was performed through an anterolateral approach, which enabled the observation of the talar dislocation ( Figs. 3A-C ). We could then reduce the talus to the ankle joint and the talonavicular joint returned to its anatomical site. Through this approach, pinning with 1.6 mm Kirschner wires was possible ( Fig. 3D ); We did not observe osteochondral injury of the talus during the surgical procedure. The ankle and syndesmal joint remained stable.

A postoperative plain radiograph showed no evidence of fracture of the talar neck or body. The patient was then evaluated 15 days after surgery. No evidence of infectious complications or wound dehiscence was observed.

**Fig. 4:**
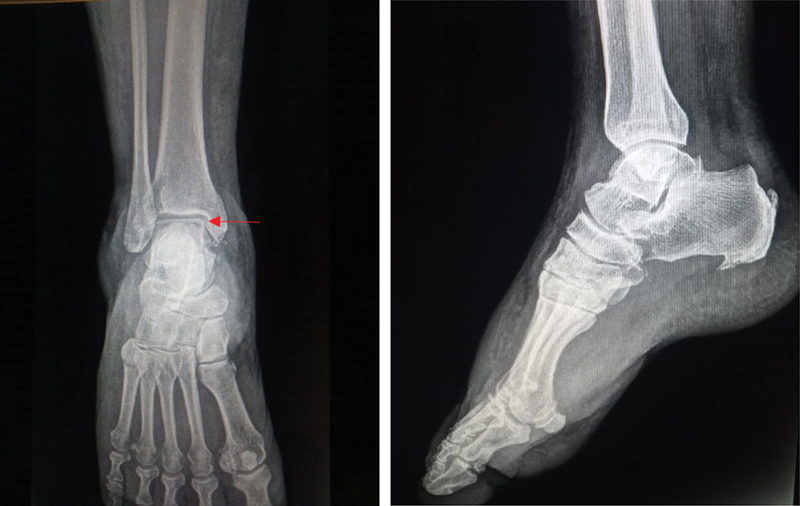
Radiographs revealed the Hawkins sign six weeks postoperatively (red arrow). No sclerosis or bone collapse of the talar dome was observed.

The nails and the splint were removed after six weeks of evolution, which was when we confirmed joint stability, and rehabilitation began for the recovery of the ranges of motion and proprioception. No weight bearing was prescribed at this time. The radiographs revealed the Hawkins sign ( Fig. 4 ).

**Fig. 5:**
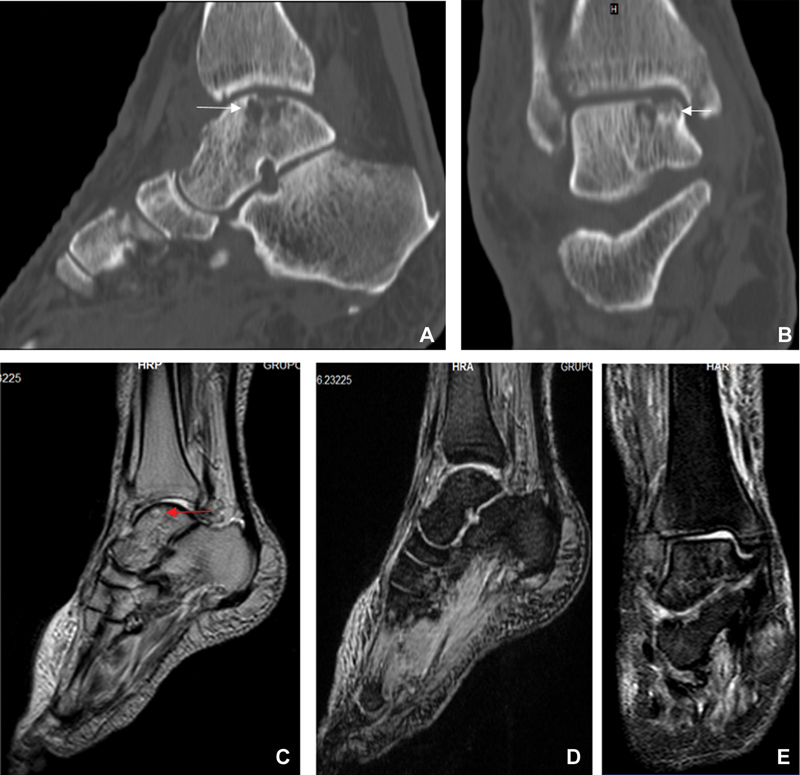
( **A,B** ) Computed tomography scans on Sagittal and coronal views performed six months postoperatively. Osteochondral fracture of the talar dome is observed (white arrows). ( **C-E** ) T2-weighted sagittal magnetic resonance imaging scan performed six months postoperatively, which shows a zone of high signal intensity in the talar dome (red arrow). Sagittal and coronal T2-weighted magnetic resonance imaging scans. No evidence of avascular necrosis or intra-articular free fragments were found.

After one month of follow-up, wound complications developed, so we started treatment with debridement, antibiotics, and daily cleaning. At six months of follow-up, the patient reported mild pain. Partial weight-bearing with a protective splint and a walker was allowed, and we observed a healed wound. Ankle motion preserved 20° of plantar flexion and 10° of dorsiflexion. Computed tomography and magnetic resonance imaging studies showed no evidence of avascular necrosis or bone collapse ( Fig. 5 ).

The case report was performed in compliance with the World Medical Association's Declaration of Helsinki on Ethical Principles for Medical Research Involving Human Subjects. The patient herein presented has given her verbal and written consent to the presentation of her health information.

## Discussion

Total talar dislocation involves the tibiotalar, talocalcaneal, and talonavicular joints. This dislocation often results from high energy trauma; it is a rare injury, especially without associated fractures, and the potential complications can seriously affect the functional capacity of patients.

Indeed, the risk of complications such as avascular necrosis of the talus, osteoarthritis, and infection may affect the course of this injury. These complications are more frequent in cases of open dislocation. Therefore, urgent closed reduction has been strongly recommended as the treatment of choice for anterolateral talar dislocation. 9 Open reduction is recommended after failed closed reduction; 7 9 however, this may increase the risk of the aforementioned complications.

Kumar et al. 10 reported a closed talar dislocation without associated fracture in a healthy 25-year-old male patient treated with closed reduction; no functional complications or avascular necrosis of the talus were described.

We herein report a case of closed dislocation of the talus without associated fractures of the talus and malleolus in a 61-year-old woman with type-II diabetes, liver cirrhosis, and a failed attempt at closed reduction. Seventy-two hours after hospital admission, open reduction was performed, due to the absence of an orthopedic surgeon to perform the surgical procedure at that time. During the surgical procedure, the main obstacle to closed reduction was the rotation of the talus.

After six months of follow-up, the patient reported mild pain and decreased range of motion in the ankle. Partial weight bearing with a protective splint and walker did not cause discomfort. We suggest that the severity of the displacement of the talus, the associated comorbidities, the delay in surgical resolution, and the damage to the surrounding soft tissues were factors that favored the development of wound complications and infection. However, the evolution has been surprising in the patient, and she currently walks without a protective splint and with minimal discomfort. She is not an obese patient and has presented adequate glycemic and liver disease control, which improves her evolution and fscilitates her return to daily life activities. Interestingly, no signs of possible development of avascular necrosis were evidenced on CT and magnetic resonance images after six months of follow-up. For the aforementioned reasons, we strongly emphasize the need for urgent treatment with closed reduction or open reduction if closed reduction has failed.

Finally, closed TTDs are infrequent injuries, especially without associated talus and ankle fractures. Early diagnosis and treatment are necessary to minimize the risk of short- and long-term sequelae. We suggest that the successful results in managing these lesions also depend on the characteristics of the lesion, the therapeutic approach, the age of the patients, and associated comorbidities.
