# Supplementary material for: Total Closed Talar Dislocation without Ankle Fracture: A Case Report
Source: Rev Bras Ortop (Sao Paulo). 2024 Apr 19;59(Suppl 1):e127–32. doi: 10.1055/s-0043-1776295 (PMC11254438; doi:10.1055/s-0043-1776295)
Supplement: Related texts

# Research article: Luxação total fechada do tálus sem fratura de tornozelo: Relato de caso

**Keywords:** articulação talocalcânea, fraturas do tornozelo/diagnóstico, luxações articulares

## Abstract

Relatamos o caso de uma mulher de 61 anos que deu entrada no pronto-socorro após cair de uma escada. A paciente foi diagnosticada com luxação talar total fechada, sem fratura do tálus ou do tornozelo. O cirurgião indicou uma redução aberta após uma tentativa frustrada de redução fechada. Aos seis meses de acompanhamento, a paciente relata dor branda e descarga parcial de peso sem desconforto; exames de tomografia computadorizada e ressonância magnética não mostraram evidências de necrose avascular ou colapso ósseo.

## Introdução

As luxações totais do tálus (LTTs) são lesões raras, que representam apenas 0,06% de todas as luxações e 2% das lesões talares. 1 A LTT é geralmente causada por um traumatismo de alta energia, e é uma lesão aberta em quase todos os casos. Weston et al. 2 relataram que 85% das luxações talares são abertas, e 15%, fechadas. Além disso, essa lesão pode ser associada com fraturas de tálus, 3 maléolo, 4 e outros ossos. No entanto, as LTTs fechadas sem fratura concomitante do maléolo e do tálus são bem menos frequentes. 5

As principais complicações que influenciam o prognóstico de LTT são necrose avascular (NAV), osteoartrite, e infecção. Isso ocorre devido ao comprometimento do suprimento sanguíneo para o tálus e os tecidos circundantes. As infecções são mais comuns após luxações abertas e a redução aberta de uma fratura talar. 6

Diversos autores 7 8 já recomendaram a redução aberta da LTT; no entanto, a redução fechada precoce de luxações fechadas do tálus tem sido enfaticamente recomendada para evitar lesões nos tecidos moles e danos à rede de suprimento sanguíneo. 9

Apresentamos o caso de uma paciente com LTT fechada com rotação e sem fratura maleolar ou talar.

## Relato de Caso

Uma mulher de 61 anos deu entrada no pronto-socorro após escorregar ao subir escadas. A paciente se queixava de dor, inchaço e deformidade no tornozelo direito. Além disso, tinha diabetes de tipo II e cirrose hepática. O exame físico revelou edema no pé e tornozelo direitos, deformidade em varo do antepé, e dor intensa. A pele apresentava um pequeno arranhão sem sangramento ativo; no entanto, não havia ferida com exposição de tecido profundo, nem evidências de comprometimento neurológico ou vascular atual ( Fig. 1 ).

**Fig. 1:** Imagem clínica que mostra o edema do tornozelo e a ferida cutânea.

Os exames radiográfico e tomográfico do tornozelo e do pé revelaram luxação anterolateral total do tálus. O tálus sofreu uma rotação medial de 90° no plano coronal, o que fez com que as facetas articulares subtalares apontassem para o maléolo lateral. Também havia uma fratura sem luxação da base do quinto metatarso. A paciente não apresentava fraturas associadas no tálus, no calcâneo ou no maléolo medial, lateral ou posterior ( Fig. 2 ).

**Fig. 2:** Radiografias simples e imagens de tomografia computadorizada que mostram a luxação talar total ( **A,B** ). As setas brancas indicam a área da articulação talocalcânea devido à rotação do tálus. A seta vermelha aponta para a fratura na base do quinto metatarso.

À admissão, houve uma tentativa de manipulação e redução fechada, mas sem sucesso. A paciente foi então internada, e uma cirurgia de redução aberta do tálus foi agendada. Este procedimento foi eficaz. A cirurgia foi realizada por via anterolateral, o que possibilitou a observação da luxação do tálus ( Figs. 3A-C ). Pudemos reduzir o tálus para a articulação do tornozelo e devolver a articulação talonavicular ao seu sítio anatômico. Essa abordagem possibilitou a fixação com fios de Kirschner de 1,6 mm ( Fig. 3D ); não observamos lesão osteocondral do tálus durante a cirurgia. O tornozelo e a sindesmose ficaram estáveis.

**Fig. 3:** ( **A-C** ) Imagens intraoperatórias. A pinça cirúrgica aponta para a área da articulação talocalcânea e realocação do tálus. ( **D-F** ) Radiografias pós-operatórias; note a fratura osteocondral na cúpula do tálus (seta vermelha).

A radiografia simples pós-operatória não mostrou evidências de fratura do colo ou do corpo do tálus. A paciente foi novamente avaliada quinze dias após a cirurgia. Não observamos nenhuma evidência de complicações infecciosas ou deiscência da ferida.

Os parafusos e a tala foram retirados após seis semanas de evolução, quando confirmamos a estabilidade articular e instituímos a reabilitação para recuperação das amplitudes de movimento e propriocepção. Nenhuma descarga de peso foi prescrita neste momento. As radiografias revelaram o sinal de Hawkins ( Fig. 4 ).

**Fig. 4:** As imagens radiográficas revelaram o sinal de Hawkins seis semanas após a cirurgia (seta vermelha). Não observamos esclerose ou colapso ósseo da cúpula talar.

Depois de um mês de acompanhamento, surgiram complicações da ferida, e iniciamos o tratamento com desbridamento, antibióticos, e limpeza diária. Aos seis meses de acompanhamento, a paciente relatou dor branda. Permitimos a sustentação parcial de peso com tala protetora e andador, e observamos a cicatrização da ferida. O movimento do tornozelo preservava 20° de flexão plantar e 10° de dorsiflexão. Exames de tomografia computadorizada e ressonância magnética não mostraram evidências de necrose avascular ou colapso ósseo ( Fig. 5 ).

**Fig. 5:** ( **A,B** ) Imagens de tomografia computadorizada sagital e coronal seis meses após a cirurgia. Note a fratura osteocondral da cúpula talar (setas brancas) ( **C-E** ). A imagem sagital de ressonância magnética ponderada em T2 seis meses após a cirurgia mostra uma zona com alta intensidade de sinal na cúpula do tálus (seta vermelha). Imagens de ressonância magnética ponderada em T2 em corte sagital e coronal. Não há evidências de necrose avascular ou de fragmentos livres no espaço intra-articular.

O relato de caso foi realizado em conformidade com a Declaração de Helsinque, da Associação Médica Mundial sobre Princípios Éticos para Pesquisa Médica Envolvendo Seres Humanos. A paciente apresentada neste caso deu seu consentimento verbal e escrito para a divulgação de suas informações de saúde.

## Discussão

A LTT acomete as articulações tibiotalar, calcaneotalar e talonavicular. De modo geral, essa luxação é provocada por traumatismos de alta energia; é uma lesão rara, principalmente na ausência de fraturas associadas, e suas possíveis complicações podem afetar a capacidade funcional dos pacientes de forma grave.

De fato, o risco de complicações como necrose avascular do tálus, osteoartrite e infecção podem afetar a evolução dessa lesão. Essas complicações são mais frequentes nas luxações abertas. Por isso, a redução fechada urgente é enfaticamente recomendada como tratamento de escolha da luxação anterolateral do tálus. 9 A redução aberta é recomendada em caso de insucesso da redução fechada, 7 9 mas pode aumentar o risco das complicações já mencionadas.

Kumar et al. 10 relataram uma luxação fechada do tálus sem fratura associada em um paciente saudável de 25 anos, do sexo masculino, submetido à redução fechada; não houve complicações funcionais ou necrose avascular do tálus.

Aqui, descrevemos o caso de uma luxação talar fechada sem fraturas do tálus e do maléolo em uma mulher de 61 anos com diabetes de tipo II, cirrose hepática, e uma tentativa fracassada de redução fechada. A redução aberta ocorreu 72 horas após a admissão hospitalar devido à ausência de um cirurgião ortopedista para realização do procedimento em um primeiro momento. Durante a cirurgia, o principal obstáculo à redução fechada foi a rotação do tálus.

Aos seis meses de acompanhamento, a paciente relatou dor branda e diminuição da amplitude de movimento no tornozelo. A sustentação parcial de peso com tala protetora e andador não causava desconforto. Sugerimos que a gravidade da luxação talar, as comorbidades, o atraso na resolução cirúrgica e o dano aos tecidos moles adjacentes favoreceram o desenvolvimento de complicações da ferida e infecção. No entanto, a evolução da paciente tem sido surpreendente; hoje, ela caminha sem tala protetora e com desconforto mínimo. Não se trata de uma paciente obesa, e seus controles glicêmicos e hepáticos são adequados, o que melhora sua evolução e facilita o retorno às atividades da vida diária. Curiosamente, não evidenciamos sinais de possível desenvolvimento de necrose avascular à tomografia computadorizada e ressonância magnética aos seis meses de acompanhamento. Pelos motivos já mencionados, enfatizamos fortemente a necessidade de tratamento urgente com redução fechada ou aberta em caso de insucesso do primeiro procedimento.

Por fim, as LTTs fechadas são lesões infrequentes, especialmente na ausência de fraturas do tálus e do tornozelo. O diagnóstico e o tratamento precoces minimizam o risco de sequelas no curto e longo prazos. Sugerimos que o sucesso no manejo dessas luxações também depende das características da lesão, da abordagem terapêutica, da idade dos pacientes, e das comorbidades associadas.
